# Microstructure, Mechanical, and Corrosion Properties of Ni-Free Austenitic Stainless Steel Prepared by Mechanical Alloying and HIPping

**DOI:** 10.3390/ma12203416

**Published:** 2019-10-18

**Authors:** Eliza Romanczuk, Krzysztof Perkowski, Zbigniew Oksiuta

**Affiliations:** 1 Faculty of Mechanical Engineering, Bialystok University of Technology, 15-351 Bialystok, Poland; z.oksiuta@pb.edu.pl; 2 Institute of Ceramics and Building Materials, 02-676 Warsaw, Poland; k.perkowski@icimb.pl

**Keywords:** nickel free austenitic stainless steel, mechanical alloying, Hot Isostatic Pressing, mechanical properties, corrosion resistance

## Abstract

An influence of the powder metallurgy route on the phase structure, mechanical properties, and corrosion resistance of Fe–18%Cr–12%Mn–N nickel-free austenitic stainless steel as a potential material for medical applications were studied. The powder was mechanically alloyed in a high purity nitrogen atmosphere for 90 h followed by Hot Isostatic Pressing at 1150 °C (1423 K) and heat treatment at 1175 °C (1423 K) for 1 h in a vacuum with furnace cooling and water quenching. More than 96% of theoretical density was obtained for the samples after Hot Isostatic Pressing that had a direct influence on the tensile strength of the tested samples (Ultimate Tensile Strength is 935 MPa) with the total elongation of 0.5%. Heat treatment did not affect the tensile strength of the tested material, however, an elongation was improved by up to 3.5%. Corrosion properties of the tested austenitic stainless steel in various stages of the manufacturing process were evaluated applying the anodic polarization measurements and compared with the austenitic 316LV stainless steel. In general, the heat treatment applied after Hot Isostatic Pressing improved the corrosion resistance. The Hot Isostatic Pressing sample shows dissolution, while heat treatment causes a passivity range, the noblest corrosion potential, and lower current density of this sample.

## 1. Introduction

In recent years, the most popular grade of the austenitic stainless steels used for medical applications has been the 316LV and an improved version of the REX 734, with the chemical composition described in the International Standards ISO 5832-1 Implants for surgery—Metallic materials, Part 1 and ISO 5832-9 Implants for surgery—Metallic materials, Part 9. Due to the low costs of production, well established manufacturing process, and good mechanical and corrosion properties, these steels are used for biomedical applications such as: implants, bone plates and screws, spinal stabilizers, cardiovascular application (stents), surgical instrumentations, and more [1]. It is also characterized by the relatively low yield to tensile strength ratio and high formability [1,2]. Some problems with medical applications of the austenitic stainless steels have been noted, such as intensive wear and pitting and fretting corrosion in the simulated body fluid environment [3,4]. This can cause premature abrupt fracture of the material, and/or release harmful products of the corrosion into the human body. The essential problem is a negative charge of the alloying ions, especially of the toxic nickel, that can be released from the steel during implantation [5,6].

Because of these mentioned problems, high manganese–nitrogen (nickel-free) stainless steel is a material to replace older generations of the austenitic stainless steels. Investigations of the influence of nitrogen on steel properties have been carried out by many authors [1,6,7,8,9]. Nitrogen in a solid solution of a steel increases the strength and improves resistance to pitting and crevice corrosion in a water solution of chloride ions [1,7]. Lim [7] reported a positive effect of nitrogen on the corrosion resistance, interstitial strengthening effect, phase stability, and also utilization of both elements, nitrogen and manganese, for partial or complete replacement of nickel for stainless steel implants. Manganese, besides its bcc lattice structure, is a strong austenite-forming element, and it can also improve the nitrogen solubility in the austenitic stainless steels.

However, there are some issues related to the incorporation of gaseous nitrogen to the stainless steel matrix during the fabrication process applying high-pressure melting technologies such as: the pressurized induction melting, the pressurized electroslag remelting, and pressurized plasma arc melting [8]. Using these methods the nitrogen concentration and gas pressure follow Sieverts’ law, where the nitrogen concentration in steel is proportional to the square root of the supply gas pressure [9,10]. Thus, the high-pressure melting technologies are complex, expensive, energy-consuming, and often difficult to control. Furthermore, the main disadvantage of these processes is limited and uneven solubility of nitrogen in the melted steel causes formation of hard nitrides locally.

Powder metallurgy (PM) is an alternative method for producing high nitrogen, nickel-free stainless steels where nitrogen can be incorporated in the solid state during the mechanical alloying process (MA) [9]. Advantages of the PM route include the relatively simple equipment and more homogeneous nitrogen distribution in the stainless steel matrix in comparison to the high-pressure melting technologies steels. Besides, the MA process is an alternative method for the high nitrogen stainless steel production, with new properties, e.g., nanostructure, absence of ferromagnetism and good corrosion resistance [11], because the number of structural defects created during high energy collisions of balls are beneficial to increase the solubility limit of nitrogen, compared to conventional melting [12,13]. Therefore, this method has been applied to manufacturing the nitrogen stainless steels by several researchers, who achieved nitriding of powder by conducting the alloying process in a nitrogen gas environment [14,15,16]. An alternative method to incorporate nitrogen into the steel matrix is the usage of nitrides, since the solubility of nitrides is higher in comparison to the solubility of nitrogen gas [11,17]. Iron, chromium, or manganese nitrides are commonly used as a nitrogen source [11,18]. Duan et al. [11,13] investigated an influence of the chromium nitride and revealed that this compound is easier to dissociate and dissolve in the Fe matrix in comparison to the more stable iron nitride. Dorofeev et al. [19] and Tsuchiyama et al. [20] found that when chromium nitride is used, 100% of austenite fcc phase was not achieved after MA of the powder, although the nitrogen amount was about 1 wt.%. It has been speculated that at some point of the MA process the dynamic equilibrium between the deformation–induced nitride dissolution and the precipitation of nano-dispersed secondary chromium nitride were achieved [19,20]. Additionally, Dorofeev [21] and Qui [22] studied the influence of chromium and manganese nitride on the α-Fe to γ-Fe transformation. Some differences in the Fe–Cr–N system under MA in the presence of the chromium nitride and manganese nitride were reported. The chromium nitride is a stable phase in the Fe–Cr–N system, while the manganese nitride is metastable. Therefore, in this work the manganese nitride was applied.

It has been shown that Mn and N also affect the deformation mechanisms of the alloy powder through the stacking fault energy (SFE) and dislocations configuration effects [23]. Lee and Choi [24] examined the SFE of the Fe–Mn binary alloy system and showed that the minimum SFE is reached when Mn concentration is about 12 wt.%, because the low SFE increases the formation of a deformation-induced α’-martensite [25,26]. Therefore, the concentration of Mn in the tested steel is adjusted for 12 wt.%.

The main goal of this work is to obtain a fully austenitic stainless steel with no Ni with the nominal composition of Fe–18%Cr–12%Mn–N (wt.%) using a mixture of elemental and manganese nitride powders by MA method followed by Hot Isostatic Pressing (HIP) and heat treatment (HT). Two different MA atmospheres and cooling rates during HT were applied to study the phase structure stability of austenitic stainless steel and the microstructure, mechanical properties and corrosion resistance of the obtained material.

## 2. Materials and Methods

### 2.1. Powders Characterization

Elemental powders of iron (average particle size ~10 μm, 99.95% purity), chromium (average particle size ~10 μm, 99.92% purity) and manganese nitride (average particle size ~60 μm, 99.95% purity) supplied by Alfa Aesar were used. The initial powder mixture with the nominal composition of Fe–18%Cr–12.8%Mn_4_N (in wt.%) was milled to obtain the Fe based alloy containing 18% Cr, 12% Mn and 0.5–1.0% N. To ensure the fcc phase stability the N content should be higher than 0.5% [1,2]. The MA process was conducted in a high–energy planetary ball mill Pulverisette 6 (Fritsch, Germany), with stainless steel balls (10 mm in diameter) and a ball to powder ratio (BRP) of 8:1. For comparison, the MA process of the Fe–18%Cr–12.8%Mn_4_N powders under argon protective atmosphere was also tested. A rotation speed of 250 rpm was chosen to not increase the temperature of the MA process. The time of MA was optimized by studying the structural evolution of the powder by means of X-ray diffractometer (XRD) Bruker Eco D8 Advance, using Cu-Kα_2_ radiation of 40 kV and 25 mA. For all samples, the angular range (2θ) of 20° to 100° with step width of 0.01 and a step time of 5 s was used. The Al_2_O_3_ sample was used as the XRD standard to correct an instrumental line broadening. A background correction the Kα_2_ stripping have been performed. Crystallite size of the powders was determined by measuring the Bragg peak width at half the maximum intensity (FWHM) and calculated using the Williamson-Hall formula [27]. This method was used to compare the obtained results with literature data. The peaks width were determined by fitting with a Pseudo-Voigt function. The lattice parameter was determined by the Nelson-Reily method [28]. Dislocation density of the milled powders was calculated using equation [29,30]:

(1) $$\Delta K≅\frac{0.89}{d}+\left(\frac{\pi Mb^{2}}{2}\right)\rho^{\frac{1}{2}}\left(K{\bar{C}}^{\frac{1}{2}}\right)+O\left(K^{2}\bar{C}\right),$$

where *K* = 2 sin*θ*/*λ*, Δ*K* = 2 cos(Δ*θ*)/*λ*, *θ* is the diffraction angle, Δ*θ* is the full width at half maximum (FWHM) of the diffraction peak, *λ* = 0.15418 nm is the wavelength of the X-rays; *d*, *ρ*, and *b* are the average grain size, dislocation density and the absolute value of the Burgers vector, respectively. *M* and *O* are constants effected by the effective outer cut-off radius of dislocations and the dislocation density. 

Powder sampling were taken at 10 h, 20 h, 40 h, 60 h, 80 h, and 90 h of MA. The morphology and particle size evolution of the MA powders were studied. After 90 h of the MA in the nitrogen atmosphere, the process was interrupted, when from the XRD analysis the phase structure of the powder was austenitic. An initial oxygen content of the preliminary mixed powders was 0.20% and after 90 h of MA increased up to 0.48% (measured by LECO TCH600 device for three specimens each), despite loading and unloading of the powders in a glove box under argon atmosphere. Particle size distribution (PSD) was measured by means of a Laser Particles Sizer Analysette 22 (Fritsch). All measurements were conducted in wet dispersion unit in range of 0.08–1000 μm. Each powder sample was dispersed in water until a proper suspension was formed, sonificated and measured three times. Usage of ultrasonic power (100 W/36 kHz) helped to obtain an optimal dispersion as it limited the presence of large powder agglomerates which can disturb the results. Prior a single measurement cycle, particles were additionally subjected to a 10-s sonification interval. 

### 2.2. Sample Preparation

The powder after MA was consolidated by two different methods: HIPping vs cold compaction and sintering. Prior to HIPping, the powder was degassed at 650 °C (923 K) for 1 h under vacuum of 10^−2^ Pa and closed in a low-carbon steel capsule. The HIPping process was performed at 1150 °C (1423 K) under an isostatic pressure of 200 MPa, for 2 h. The heating rate was 400 °C/h (673 K/h), whereas the cooling rate was 720 °C/h (993 K/h). The HIPped material was annealed at 1175 °C (1448 K) for 1 h in a vacuum with furnace cooling (denoted as HIP+HT–FC) or water quenching (denoted as: HIP+HT–WC), to reduce residual stress of the material after HIPping. For comparison, the mechanically alloyed powder was cold compacted in a cylindrical die of 20 mm in diameter and 10 mm high, using an uniaxial hydraulic press with a pressure of 400–600 MPa and sintered at 1150 °C (1423 K) and 1200 °C (1473 K) for 2 h, in nitrogen with cooling rate of 600 °C/h (873 K/h). No lubricant or process controlled agent was applied. 

### 2.3. Characterization of Consolidated Material

#### 2.3.1. Microstructural Characterization

The density of the consolidated samples was measured by the Archimedes method. For better understanding of the sintering process and mass reduction related to it, a densification parameter ψ was calculated using Equation (1) [31]:

(2) $$\psi =\frac{\rho_{s}-\rho_{g}}{\rho_{t}-\rho_{g}}\times 100\%,$$

where: *ρ_g_* is the green density (kg/m^3^), *ρ_t_* is the theoretical density, and *ρ_s_* is the density of the sample after sintering. The densification parameter (ψ) was used to indicate an ability of the Fe–18%Cr–12%Mn–N compacts to be densified (shrinkage) during sintering. 

For metallographic investigation, the samples after HIPping and sintering, were polished, etched and observed using Optical Microscope (OM, Olympus GX41, Tokyo, Japan) and Scanning Electron Microscope (SEM, Hitachi 3000N, Tokyo, Japan) equipped with an Energy Dispersive Spectrometer (EDS). The electrolytic etching for 15 s at 3 V in 10% of oxalic acid was performed. The grain size of the tested specimens was examined according to ISO 643:2003 [32]. Chemical analysis of the consolidated specimens was performed by LECO TCH600 analyzer (Leco, St Joseph, MI, USA), Spark Spectrometer Thermo ARL Quantris (Thermo Fisher Scientific, Switzerland) and Scanning Electron Microscope equipped with an Energy Dispersive Spectrometer (SEM-EDS) analysis (Hitachi 3000N, Tokyo, Japan).

#### 2.3.2. Mechanical Properties

**Figure 1:**
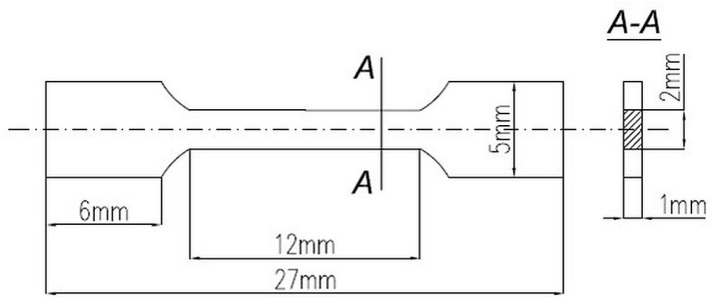
The tensile test specimen.

The Vickers hardness (HV10) of the samples was measured by using the Anova Hardness Tester (Innovatest, Maastricht, The Netherlands) under a load of 98 N (10 kg) and an indentation time of 10 s. The microhardness (µHV0.2) tests were performed using the PMT-3 tester under a load of 1.96 N (0.2 kg) for 10 s. The mechanical properties of the steel at room temperature were determined using a tensile testing machine MTS 858 with an extensometer 3542 Epsilon, with crosshead speed of 1 mm/min, according to EN ISO 6892–1:2011 [33]. Tensile specimen is shown in Figure 1. Five test specimens from the HIPping and heat treatment materials were tested. Fracture surface was observed by the SEM.

#### 2.3.3. Corrosion Tests

Corrosion resistance of the HIPped and heat treated with furnace cooling samples (HIP+HT–FC) and 316LV as a reference specimens was tested according ISO 10993–15 [34]. The PGP201 VoltaLab galvanostat/potentiostat (Radiometer Analytical, France) equipped with the VoltaMaster 4 software was used. As a corrosion medium, 70 mL of the Hank’s solution was used.

An open corrosion potential (*E_OCP_*) and potentiodynamic corrosion tests were carried out. A three-electrode electrochemical measuring circuit was adapted in each corrosion test. The reference electrode was a saturated calomel electrode (SCE) with redox potential of +0.242 V vs. a standard hydrogen electrode (SHE) at 25 °C. An auxiliary electrode was a platinum plate with a contact area of 31.4 mm^2^. The contact area of the tested samples was 64 mm^2^. 

Prior to corrosion testing five samples were mechanically polished with SiC abrasive papers up to 2500, then polished using 0.02 µm alumina suspension, degreased in ethanol and rinsed with distilled water. The corrosion tests were repeated five times, to ensure reasonable reproducibility.

The corrosion process was characterized by several electrochemical quantities:

(a) the open circuit potential (*E_OCP_*), recorded for 2 h with the sample immersed in the Hank’s electrolyte;
(b) the polarization resistance (*R_p_*), calculated from traces of the polarization curve at 30 mV versus *E_OCP_*;
(c) the Tafel slopes (*b_a_* and *b_c_*) and the corrosion current (*I_corr_*) using the Stern-Geary [35] Equation (2):
  (3) $$I_{corr}=\frac{b_{a}b_{c}}{2.3R_{p}\left(b_{a}+b_{c}\right)}$$

The Tafel slopes were calculated from plots of the polarization curves at ±200 mV versus *E_OCP_*.

Potentiodynamic polarization curves were obtained with a scan rate of 3 mV/s in the potential range.

The surface of the samples after polarization in the Hank’s solution was observed by means of the optical microscope.

## 3. Results

### 3.1. Characterization of the Mechanically Alloyed Powders

**Figure 2:**
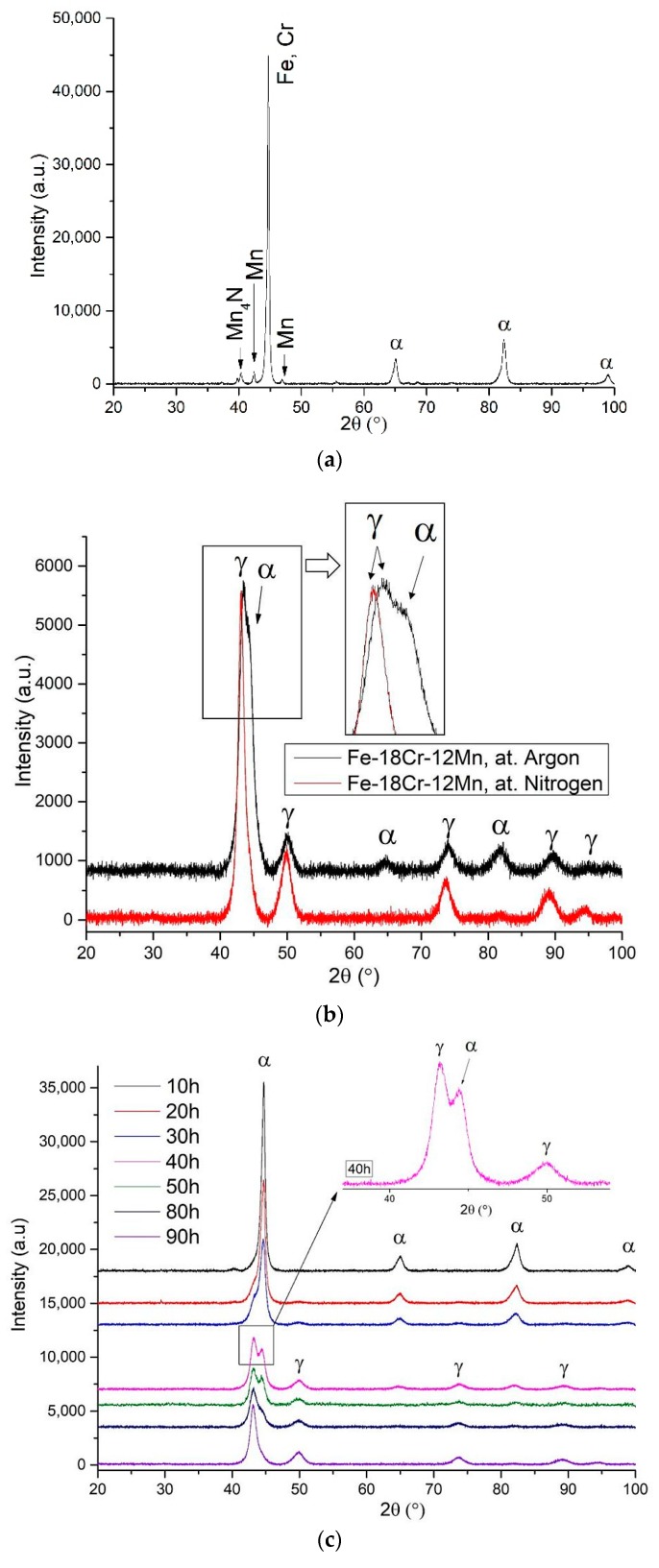

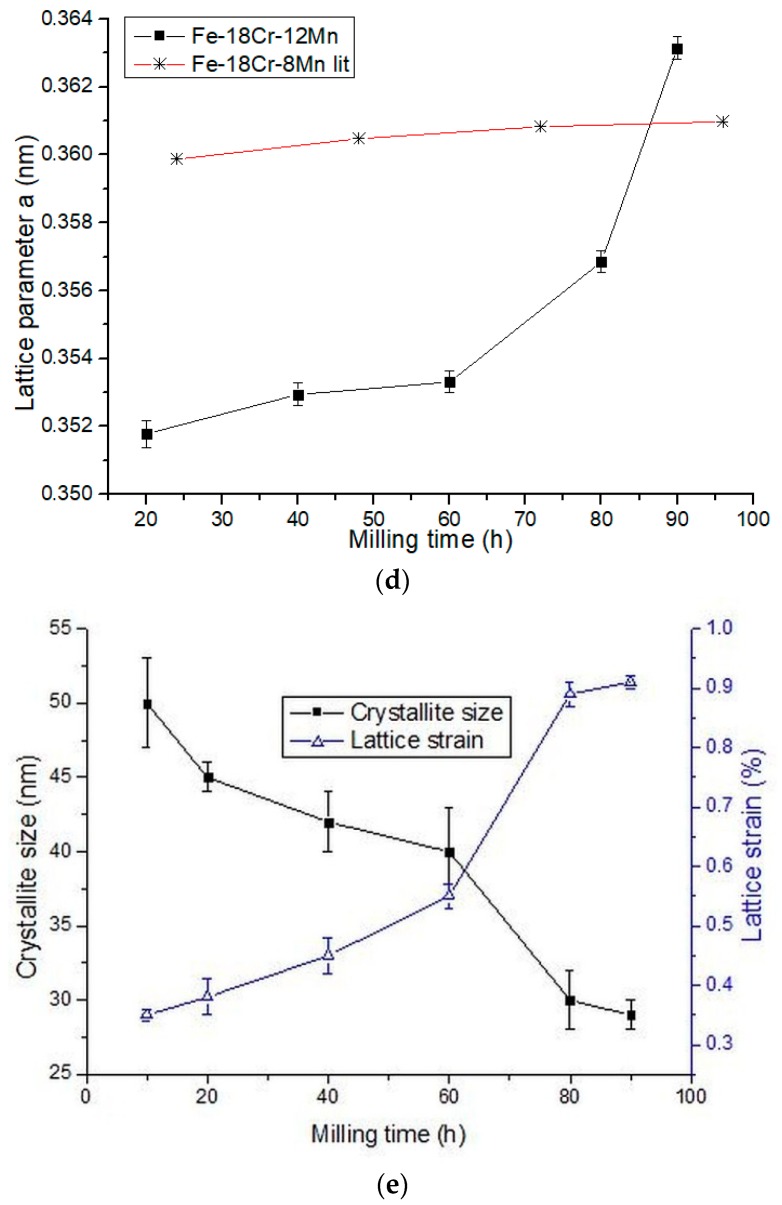
X-ray diffractometry (XRD) data of the Fe–18%Cr–12.8%Mn_4_N powders: (**a**) powders mixture in nitrogen for 1 h, (**b**) both powders mechanical alloying process (MA) in nitrogen and argon for 90 h, (**c**) MA for various milling times under nitrogen up to 90 h, (**d**) lattice parameter of the powder MA under nitrogen and the literature data for Fe–18%Cr–8%Mn powder (elemental powders of Fe, Cr, and Mn) MA under nitrogen [38], (**e**) crystallite size and lattice strain of as-milled powder under nitrogen atmosphere.

XRD pattern of the initial powders mixture after 1 h of ball milling is shown in Figure 2a. The powder composition shows peaks of Fe, Cr, Mn, and Mn_4_N. Differences in the XRD curves of powders after 90 h of milling under argon vs. nitrogen are shown in Figure 2b. In the powder MA in argon the peaks of both austenitic and ferritic phases were detected. This indicates that with the MA process parameters applied in this work, it is difficult to obtain a fully austenitic phase structure using argon atmosphere. Relatively high nitrogen content measured in the powder after MA, about 1%, as presented in Table 1, ensured the austenitic structure. The same tendency was reported by Haghir et al. [14] and Salahinejad et al. [36]. Therefore, the powder mechanically alloyed in nitrogen was chosen for further investigation.

XRD patterns of the powder after different time of MA in nitrogen is presented in Figure 2c. At 10 h of milling the main peaks of ferrite (α-Fe) was observed. The diffraction peaks of γ-Fe appeared after 20 h of MA. The α-Fe peaks intensity decreased gradually, while the γ-Fe peaks became stronger. Two-phase structure was observed (see enlarged XRD main peak after 40 h in Figure 2c) from 40 to 80 h of ball milling. However, after 90 h, the only peaks from austenite were detected. A similar trend was reported by Cisneros et al., where after 80 and 96 h of MA fully austenitic structure was confirmed by XRD analysis [37].

A closer analysis of the XRD profiles suggests that with prolonging the milling time the main peaks become broader and their positions shift toward lower diffraction angles. The peak broadening is due to reduction in the crystallite size. The crystallite size decreased with prolonging the milling time from 100 ± 6 nm after 1 h of MA, to 29 ± 2 nm after 90 h. After 60 h of milling the crystallite size significantly decreased (Figure 2e). Duan et al. [11] calculated the crystallite size by the Williamson-Hall method, and the XRD pattern showed the only peaks from the austenite. The crystallite size was about 14.5 nm, which was twice lower in comparison to this work. However, Duan et al. [11] used different MA parameters, e.g. 350 rpm, BRP 10:1, different diameter of milling balls (ø 6 mm, ø 8 mm, and ø 10 mm) and nanosized chromium nitride powder that affect the crystallite size.

In Haghir et al.’s [14] work the crystallite size was ~9 nm for powders milled in the nitrogen atmosphere. The authors also investigated an influence of the crystallite size on the α → γ transformation. They suggested that for the Fe–18%Cr–11%Mn system, the critical crystallite size required to get stable fcc phase is about 5–10 nm. However, the results presented in this work indicate that the crystallite size of 29 ± 2 nm is also sufficient to obtain the austenitic phase, supported by the Mn_4_N compound powder. 

Furthermore, with increasing the milling time, the lattice strain after 90 h of MA increased up to 0.91 ± 0.02 and the dislocation density, calculated from Equation (1), was 1.9 ± 0.8 × 10^15^ m^−2^ and 9.3 ± 2.4 × 10^15^ m^−2^ at the initial and final stages of the MA process, respectively. Note that the lattice strain is higher in comparison to the results presented by Amini et al. [39] where an amorphous phase was detected. It is well known that the lattice strain increases with increasing the dislocation density [39].

Figure 2d displays an expansion of a lattice parameter of the powder MA in nitrogen as a function of milling time. The lattice parameter constantly increases with increased milling time, contrary to the data reported by Salahinejad et al. [38], in which this parameter is almost unchanged. An increase in a lattice parameter is usually caused by the solubility of substitutional and interstitial alloying atoms [38]. These dissimilarities in the lattice parameter may be due to differences in chemical composition, parameters of MA process and initial powders used. Note that an abrupt increase in this parameter is observed between 60 to 90 h of MA that can be related to alteration of the manganese nitride powder solubility in the steel matrix (Figure 2d). 

It is assumed that during the initial stage of MA, the relatively hard and stable Mn_4_N particles may constantly reduce in size but not necessarily dissolve in the steel matrix. After 60 h of MA, however, the dissolution process of the manganese nitride activates, increasing the lattice parameter and nitrogen content in the powder. This can be confirmed by the interplanar d-spacing data of the powder and nitrogen content presented in Table 1, where after 60 h of MA both parameters suddenly increased.

**Figure 3:**
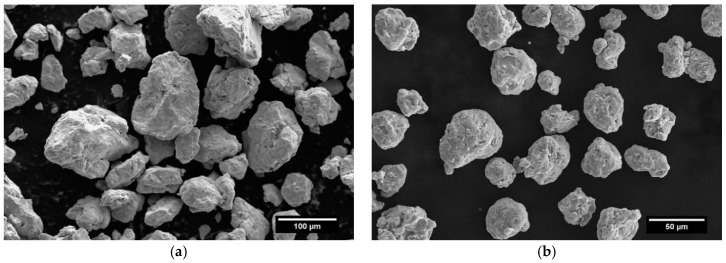
Scanning Electron Microscope (SEM) images of the powder after (**a**) 1 h and (**b**) 90 h of MA in nitrogen.

The MA process significantly changed a morphology of the Fe–18%Cr–12.8%Mn powder. Note, that the atmosphere of MA process (Ar or N_2_) has no influence on the morphology of both powders. Figure 3 shows SEM images of the powder particles after 1 h (Figure 3a) and 90 h of MA in nitrogen (Figure 3b), respectively. At the beginning of the MA process, a bimodal particle distribution of the powder was observed with irregular and aggregated shape. However, at the end of the process the particles were more regular and rounded with a smoother surface.

**Figure 4:**
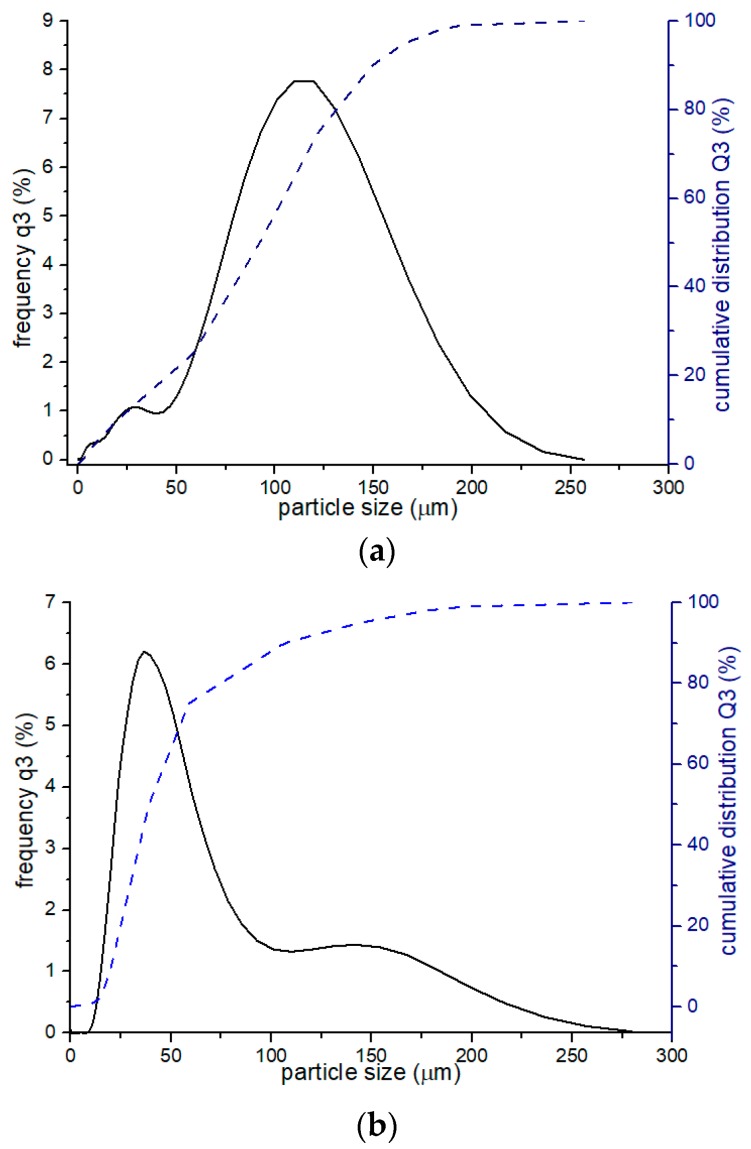
Granulometric analysis of the powders after (**a**) 1 h and (**b**) 90 h of MA.

The laser analysis of the particles size after 1 h and 90 h of MA is presented in Figure 4 and in Table 2, in which the statistic parameters of the powder particles are shown.

For powders after 1 h and 90 h of milling, the volume mean diameter (D[4,3]), median (D50) and Mode (Mo) were determined. After 90 h of MA, the average powder particle size D[4,3] is about 52 µm. The curve in Figure 4b is asymmetric and irregularly distributed. After 1 h of MA (Figure 4a) the mean particle size is higher than after 90 h of milling, which anticipates that at the beginning a welding process dominates. 

The particle size has an impact on the consolidation process, influencing the density and therefore final material properties. The smaller the particle size, the better mechanical properties of the sintered material. Usually after accomplishing MA process, particle size should be equal or lower than an initial particles size used for analysis [40]. In this work, after 90 h of MA the average particle size is about 13% lower than an initial size of the Mn_4_N powder, however, it is not as refined as the initial Fe and Cr powders.

### 3.2. Cold Compaction and Sintering of the Powder

It was difficult to obtain a good quality of green compacts after cold compaction of the highly deformed mechanically alloyed particles under a pressure lower than 600 MPa without cracks or particles’ detaching. Particles’ morphology, hardness, and compression pressure influence densification process of green compacts. The compaction pressure of 600 MPa ensured specimens with no cracks or delamination, however, having a theoretical density of 76% ± 4%.

Sintering at 1150 °C (1423 K) and 1200 °C (1473 K) for 2 h in nitrogen caused the density increment of ~9% and ~12%, respectively (see Table 3). The nitrogen was chosen to prevent the loss of this element during sintering and to ensure a fully austenitic phase structure of the sinters. Duan et al. [11] measured the nitrogen content before and after sintering in nitrogen with the conclusion that despite the nitrogen atmosphere used, the amount of the nitrogen may slightly decrease.

The densification parameter of the sintered samples at 1150 °C (1423 K) and 1200 °C (1473 K) was calculated at 25% and 33%, respectively. As expected, the green compacts sintered at 1200 °C had a higher densification ratio due to the higher shrinkage and oxygen reduction, similar to the references reported in [11,41].

**Figure 5:**
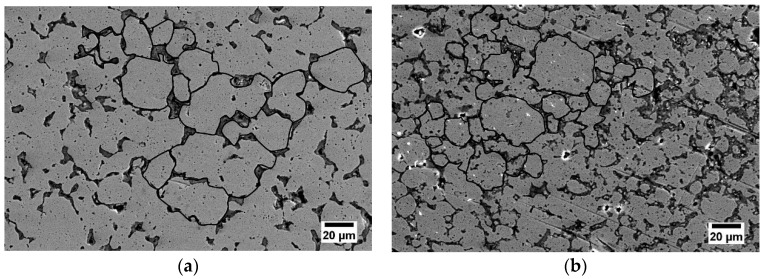
SEM images of the sintered austenitic stainless steel at: (**a**) 1150 °C and (**b**) 1200 °C.

**Figure 6:**
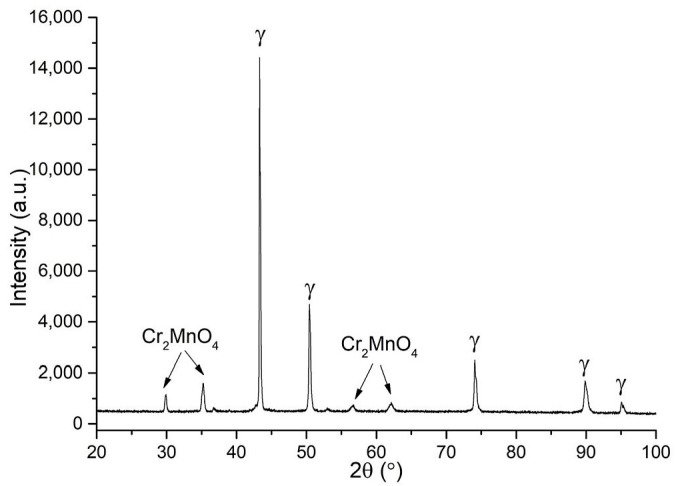
XRD patterns of the sintered material at 1200 °C in nitrogen.

Microstructure of the samples after sintering is shown in Figure 5a,b, however, it was difficult to etch the porous sample after sintering, thus the prior particles are visible, surrounded by the pores and precipitations, with an average particle size of about 25 μm. In both samples irregular pores and complex oxide precipitations was observed, as confirmed by the XRD data (Figure 6).

The microhardness of both sinters is slightly higher in comparison with the hardness value, (see Table 3), due to a porosity factor. However, higher sintering temperature caused an increase in the hardness but a decrease in the microhardness of the material. This can be related to the annealing process that took place at the higher temperature. On one hand it caused a recrystallization process but on the other hand increased the density that affected hardness. The microhardness of the sintered samples is almost twice as high as in literature [41], probably due to the Mn_4_N powder used during the MA process.

### 3.3. Characterization of HIPped Material

Bulk chemical composition of all tested specimens is shown in Table 4. The composition of the HIP and HT steel can be assumed as Fe–18%Cr–12%Mn–0.5%N (in wt.%). Note that the vacuum heat treatment of the steel decreases the nitrogen content of ~45%. Since oxygen and carbon content also decreases, it can be assumed that during HT complex chemical reactions take place that caused a depletion of these elements.

**Figure 7:**
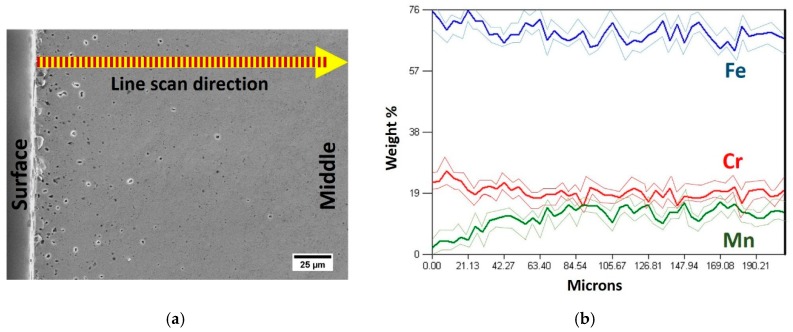
(**a**) Scanning Electron Microscope (SEM) image with an Energy Dispersive Spectroscopy (EDS) analysis of the cross-section of HIP+HT–FC sample; (**b**) EDS line scan of main elements.

Note that in the HIP+HT–FC material the Mn content fluctuates with the distance from the surface of the sample to the core (see Figure 7). An ~3.5% lower manganese content on the surface of the tested material after furnace cooling is related to a vapor pressure of this element during prolonged HT process. Salak reported [42] that Mn has significantly higher vapor pressure at 1200 °C (~94.69 Pa) than such alloying elements as Fe and Cr, 8.40 × 10^−3^ and 3.13 × 10^−2^ Pa, respectively. Therefore, a vacuum sintering atmosphere can simultaneously reduce oxygen and carbon content, but also contribute to an evaporation of manganese from a surface of samples. This explains the Mn loss and porosity observed near the surface.

**Figure 8:**
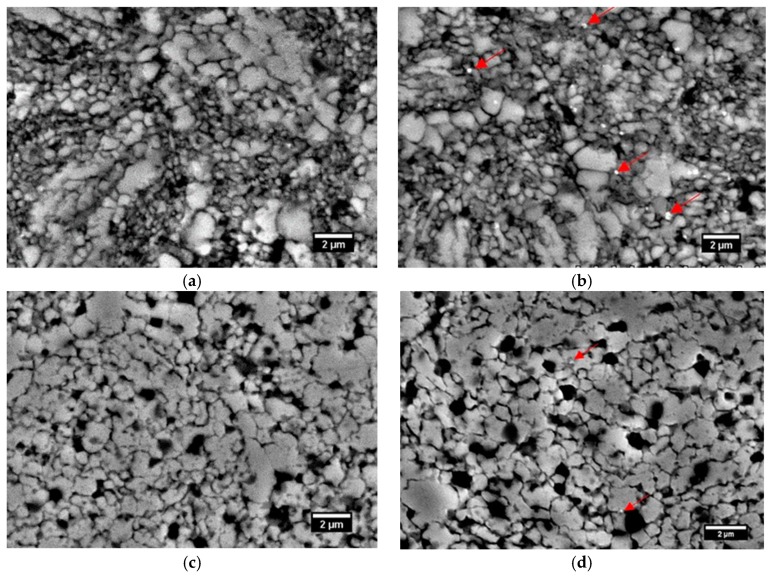
SEM images of the HIPped and HT sample at 1175 °C: (**a**,**b**) as-HIPed, (**c**,**d**) HIP+HT and water cooled.

In Table 5, the density, microhardness, and grain size of the HIPped materials are presented. The specimens after HIPping have 10% higher density than the sintered materials. Heat treatment in vacuum caused slight changes in the density, hardness, and grain size in comparison to the as-HIPped state. A minor increase in the density after furnace cooling might be related to the reduction of oxygen content. It caused an increase of the interparticle (neck) contact area and a more intensive densification process [43]. The slightly lower density of the water cooled sample is difficult to explain, however, it may be related to the local martensitic transformation that may occur after fast cooling [44]. The heat treatment of the HIPped samples caused the grain growth (see Figure 8). The microstructure of the HIP sample has relatively fine grains with an average grain size of 1.76 ± 0.35 μm. However, 75% of the grain size is lower than 5 μm and 25% is coarser ~20 μm. Bright small dots marked by red arrows in Figure 8b indicate fine chromium carbide–nitrides. A typical microstructure after HT is presented in Figure 8c,d. It exhibits a refined microstructure with local grain growth caused by a recrystallization process.

**Figure 9:**
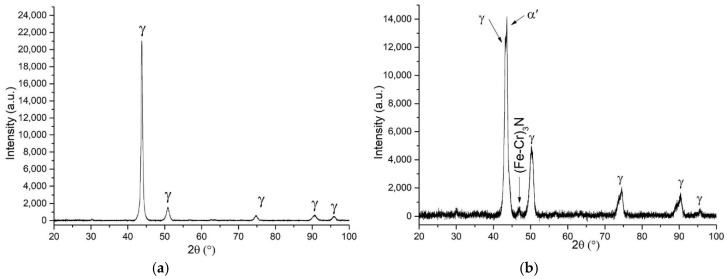
XRD patterns of the tested steel: (**a**) as-HIPped (**b**) HIPped and water quenched.

As expected, the microhardness of the HIPped sample is 40% higher than the sintered materials (see Table 4 and Table 5), and heat treatment changed the microhardness of as-HIPped steel. The sample cooled with furnace had lower microhardness in comparison to the water quenching sample. These differences are caused by the appearance of the martensite confirmed by the XRD data presented in Figure 9b. The material after HIPping has the austenitic phase structure (Figure 9a). Rapid quenching from austenite to room temperature might result in a local formation of a martensite with a very hard structure, despite a relatively low carbon content measured in the steel (see Table 4) [45]. Also, the XRD analysis revealed (Fe–Cr)_3_N precipitations that may have some contribution to the hardness of the HIP+HT–WC steel.

A literature review of the hardness of nickel-free austenitic steels gives values varying from 350 HV (3432 MPa) to 550 HV (5394 MPa) depending on the manufacturing route, material composition, density, and microstructure [8,46,47]. Results presented here show the influence of cooling rate on the steel properties. Lower hardness measured for the HIP+HT–FC steel can be explained on the basis of the recrystallization phenomenon that took place at higher heat treatment temperature (1175 °C), and can be confirmed by the grain size measurements (see Table 5). 

### 3.4. Mechanical Properties

**Figure 10:**
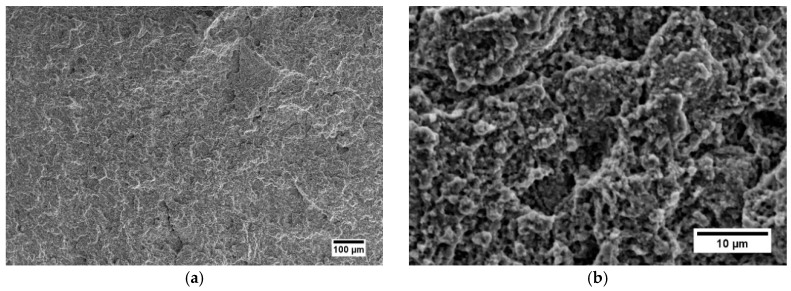

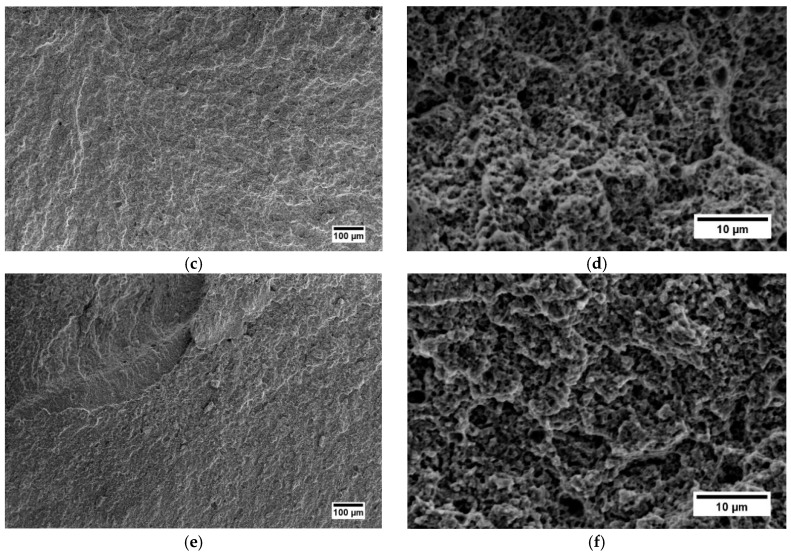
SEM micrographs of the fracture surfaces of: (**a**,**b**) as-HIPped, (**c**,**d**) HIPped and furnace cooling, (**e**,**f**) HIPped and water cooling specimens after tensile tests.

**Figure 11:**
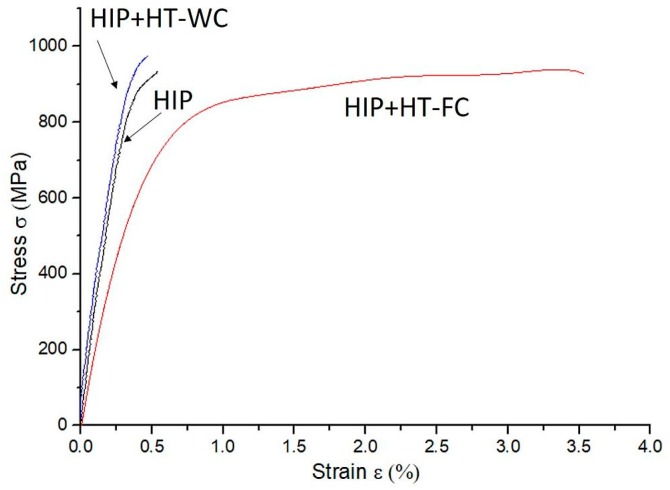
Tensile stress–strain curves for HIPped, HIP+HT–WC, and HIP+HT–FC samples.

Mechanical properties of the steel after tensile testing are summarized in Table 6. An average tensile strength was obtained for both the as-HIPped and HIP+HT–FC samples, 934 ± 11 and 938 ± 8 MPa, respectively. The HIP+HT–WC sample has about 5% higher ultimate tensile strength (975 ± 10 MPa). Higher microhardness values and very low ductility of both as-HIPped and HIP+HT–WC samples, supported by surface fracture observations (Figure 10a,b,e,f) proves that this material is brittle. The furnace cooling heat treatment does not affect the tensile strength, but the elongation (*ε_t_*) increases up to 3.51% ± 0.13% (see Figure 11). The ductile fracture of the HIP+HT–FC sample is observed (Figure 10d). This material has ~30% higher an ultimate tensile strength (UTS) of 650 MPa in comparison to results presented in [40], where the HIPping process was also applied. The Young modulus of the tested steel is relatively lower, due to the porosity.

In [48,49], the UTS was in the range of 960–985 MPa, similar to those presented here, however, the elongation was 48–52%. This significant elongation was achieved by applying after HIPping an additional thermo-mechanical treatment (hot rolling). Hence, to improve mechanical properties of the HIPped stainless steel, further thermo-mechanical treatments have to be conducted.

### 3.5. Corrosion Properties

**Figure 12:**
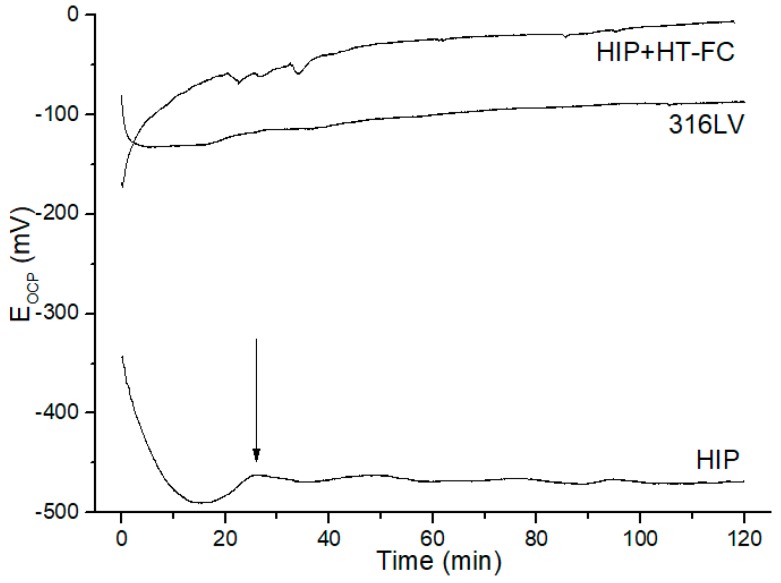
Open circuit potential versus time curves measured in Hank’s solution for as HIPped, HIP+HT–FC specimens, and 316LV steel (as reference material).

An open-circuit potential of the HIPped and HIP+HT–FC specimens was measured in Figure 11. It indicates deviation in the open-circuit potential of the as-HIPped, HIP+HT–FC and the 316LV steels. The surface of the HIPped sample is more active thus, initially, the potential decreased getting a minimum value, and then slightly increased (arrow in Figure 12). After 25 min of the process the potential did not change significantly and stabilized. The decrease in the *E_OCP_* is attributed to removal of the air-formed oxide film from the polished surface. It has been reported that this type of film is an iron oxide and might be diluted in corrosive electrolytes, even in the open-circuit condition, either by direct dissolution or via undermining of a metal dissolution if the film is not continuous [50,51,52]. A similar tendency is observed for the 316LV steel, however, with a higher potential. In the 316LV the *E_OCP_* stabilizes after ~40 min, comparing with the potential at t = 0. It can be concluded that the film on the surface of the HIP+HT–FC sample is more stable than in the as-HIPped and 316LV specimens.

**Figure 13:**
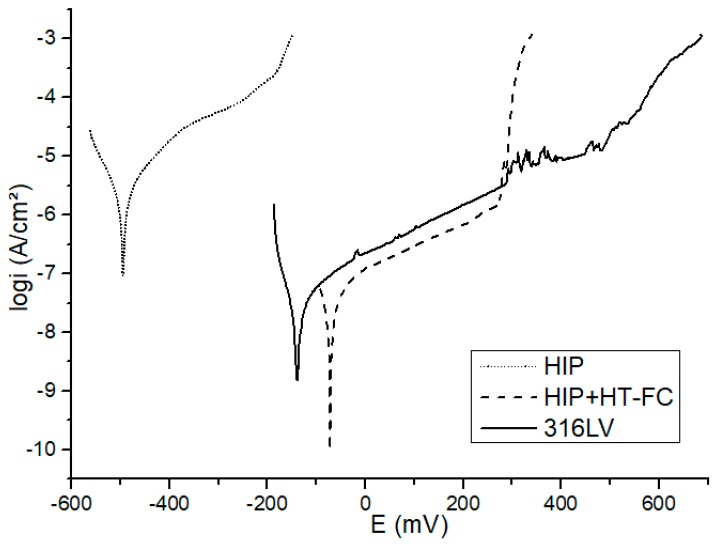
Potentiodynamic polarization curves of the tested specimens in Hank’s solution.

After immersion of the specimens in Hank’s solution for 2 h and the attainment of steady-state conditions, anodic potentiodynamic polarization scans were carried out (Figure 13). The formation and stability of the protective passive film can be realized from the passive current density and passive range. The anodic curve is different for the HIPped and HIP+HT–FC samples. The HIPped sample shows dissolution, while heat treatment causes a passivity range, the noblest corrosion potential and lower current density. It should be recalled here that the sample after heat treatment and furnace cooling has about 3.5% less manganese content at the surface, which affects the passive layer and increases the corrosion resistance [51,53,54]. Besides, chromium-rich precipitations also reduces the corrosion resistance. The potentiodynamic curve of the 316LV is similar to the HIP+HT–FC sample, and the range of the passivation region is similar too, but the *E_cor_* is lower in the latter in comparison to the 316LV (see Table 7). 

The polarization resistance (*R_p_*) parameter summarized in Table 7 is higher for the HIP+HT–FC sample in comparison with the as-HIPped material, but it is lower than commercial 316LV steel. The *R_p_* is representative for the degree of protection of the passive layer of the steel surface. The higher the *R_p_* value, the better the corrosion resistance [55,56].

The Tafel slopes (*b_a_* and *b_c_*) were determined by fitting a theoretical polarization curve to the experimental polarization curve plotted in a range of ±200 mV versus *E_OCP_*. The corrosion current (*I_cor_*) shows a degree of degradation of the alloys. An alloy with a tendency toward passivation will have a greater value of *b_a_* than *b_c_*, whereas the alloy that corrodes will have revers tendency [56,57]. The higher *b_a_* than *b_c_* values for all tested samples indicate an anodic control in the corrosion process. This implies an existence of a passive layer on the surface of the tested materials. The oxide layer on the alloys gives rise to a typical passive state with a low corrosion current density, as presented in Table 7 for the HIP+HT–FC sample.

**Figure 14:**
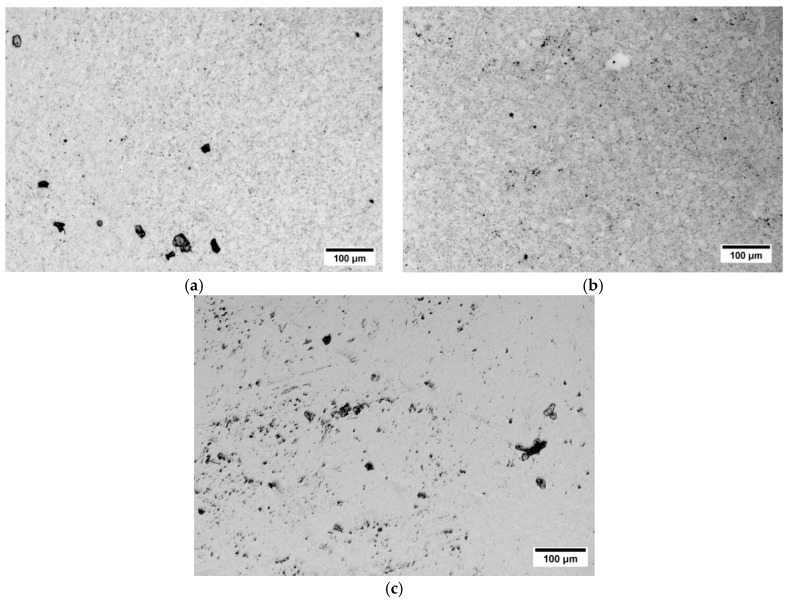
Optical Microscope (OM) images of the surface of specimens: (**a**) as-HIP, (**b**) HIP+HT–FC, and (**c**) 316LV steel after the polarization test in the Hank’s solution.

Typical surface morphology of the tested samples after polarization tests is shown in Figure 14. After polarization testing the as-HIPped sample has large corrosion pits of 10–35 µm. In the HIP+HT–FC sample relatively small corrosion pits and pores were observed. In the 316LV steel, the pits were bigger, more irregular in shape, and densely covered the surface which confirms the corrosion properties of this material.

## 4. Discussion

In this work, the Fe–18%Cr–12%Mn–N steel was prepared by the powder metallurgy route using elemental Fe, Cr, and Mn_4_N compound powders and nitrogen as MA atmosphere, then consolidated using two different methods: sintering under nitrogen and HIPping. It was found that this steel has ductility and is sensitive for a cooling rate during HT.

The fcc phase structure was detected by XRD after 90 h of MA in nitrogen in the steel powder with Mn_4_N compound addition, despite the fact that the MA process parameters were not high. This can also be confirmed by the interplanar d-spacing data of the powder, presented in Table 1, where after 80 h of MA this parameter increased suddenly. Furthermore, after 80 h of milling the amount of nitrogen also increased significantly and at the end of the ball milling (after 90 h) it was ~1.0%, which is about 35% higher in comparison to [14], where similar process parameters were used. Simultaneous addition of nitrogen in two forms (as powder and atmosphere) may explain the phenomenon of an abrupt increase in the lattice parameter and interplanar spacing. 

Obtainment of 100% austenite is a very crucial requirement during the production of austenitic stainless steel, especially for medical applications. Salahinejad et al. [18] investigated by XRD an influence of various annealing temperatures 1000–1300 °C of the as-milled powders followed by water quenching on the stability of the fcc phase structure. The main conclusion was that a fully austenitic structure was obtained after water quenching of the powder from the temperature of 1150–1250 °C.

The fcc phase structure stability derives from the austenite stabilizing elements Mn, N, and C and microstructure refinement [1,58]. In this work, the XRD analysis of the bulk material after annealing at 1175 °C and water quenching revealed both austenitic and martensitic phases. One of the possible explanations is that after water quenching inhomogeneous concentration of carbon can cause undesired local austenitic–martensitic phase transformation that increases hardness and decreases ductility and corrosion properties of the material. 

It is well known that the HIP method allows one to obtain a material with 100% of the theoretical density, however, only under certain conditions. It is difficult to obtain a fully dense HIPped steel if the powder shape is not spherical and soft, and is not densely packed in the capsule. Also, oxygen content of the powder should be as low as possible, usually below 0.2%. This also affects the HIPping process as well as mechanical properties. Annealing of the milled powder, prior to HIPping, in reducing atmosphere, e.g., hydrogen, vacuum, or mixture of hydrogen and nitrogen, could reduce the oxygen content and decrease the hardness. However, the issue is that annealing at higher temperature triggers sintering of the very defected powders that complicates the HIPping process because the air is closed in the pores and the sintered necks should be destroyed to increase the density and mechanical properties, especially ductility. Thus, further thermo-mechanical treatment has to be applied.

For the sintering process, a nitrogen atmosphere was chosen. This is not a reducing atmosphere and retards the sintering process due to the small atomic radius of nitrogen that occupies interstitial sites and promotes an increase in atomic packing. This decreases the atomic diffusion coefficient and, therefore, influences the density of sintered materials [41]. On the other hand, the nitrogen atmosphere can only provide required nitrogen content in the steel that ensures a fully austenitic phase structure. However, during sintering, nitrogen atoms escape from a steel matrix when the temperature reaches a high level. Meanwhile, nitrogen atoms from the nitrogen atmosphere continuously diffuse into a matrix, which prevents the loss of large quantities of nitrogen. However, the dissociation of nitrogen molecules and the diffusion of nitrogen atoms into the Fe-matrix is more difficult than the process of nitrogen atoms running out of the matrix at such high annealing temperature. Therefore, a part of the nitrogen loss is inevitable [11].

The heat treatment affects the mechanical properties of the as-HIPped samples. The HIPped and HIP+HT–WC specimens have higher hardness and a very low ductility. The low ductility of the HIP+HT–WC sample can be caused by the austenite to martensite phase transformation. This may suggest that the amount of the austenite stabilizing elements (Mn and N) was not enough in the steel or carbon content has to be reduced to obtain a fully fcc phase structure. 

It can be assumed that during the vacuum heat treatment of the as-HIPped steel, a complex chemical reaction takes place that causes the depletion of the alloying elements. As mentioned before, manganese sublimates at relatively low temperatures, and its partial pressure reaches about 19.88 Pa at 1100 °C [42]. The manganese vapor then reacts with oxygen according to the reaction:

2Mn_(gas)_ + O_2_ → 2MnO (4)

If the sintering process is performed in an atmosphere with low vacuum pressure, then the reaction between Mn_(gas)_ and oxygen occurs at greater distances from a surface of specimens. At higher oxygen partial pressure, the oxidation of manganese will be more extensive and include the formation of a green MnO film on the surface of parts, which is transported away by the vacuum atmosphere from the work space (tube of sintering furnaces) [59,60]. If MnO is not removed from the surface, it can be reduced to Mn with carbon at a temperature over 1100 °C, through the Mn−C (Mn_5_C_2_) reduction reactions as follows [42]:

5MnO + 7C → Mn_5_C_2_ + 5CO_(g)_ (5)

2MnO + Mn_5_C_2_ → 7Mn_(g)_ + 2CO (6)

As the corrosion resistance results show, the highest open-circuit potential was observed for the heat treated and furnace cooled sample. The theory of the ennoblement of passive metals upon prolonged exposure under open-circuit conditions is now well-developed and it shows that a positive drift in the *E_ocp_* occurs because of a progressive thickening of the barrier oxide layer [51,61].

The HIP+HT–FC sample shows the best corrosion potential and lower current density, probably due to Mn surface depletion (~3.5% less Mn in comparison to the HIPped sample). Manganese negatively affects the characteristics of the passive layer, which is attributed to its strong chemical activity. The electronegative of Mn (1.55) is lower than iron (Fe) (1.83), suggesting the ability of Mn to attract electrons is weaker than that of Fe. Mn-rich areas or Mn-rich phases in stainless steels easily lose their electrons and become anodes in an electrochemical reaction system in aggressive solutions. This promotes the anodic dissolution of stainless steels. Furthermore, the range of pH value and potential that Mn^2+^ is stable on the potential–pH plot is much wider than that of Fe^2+^, indicating that Mn is more active than Fe, and tends to become Mn^2+^ in corrosive environments. In addition, Mn hardly improves the electrode potential of Fe-based solid solutions [53,54]. The Mn-rich oxides are less protective. Moreover, some researchers noted that, high Mn alloying content has a detrimental influence on pitting corrosion of stainless steels, even though the nitrogen solubility could be significantly raised [7,62].

As it is known, in Hank’s solution there are compounds with chloride ions. These ions react with the metals according to the following reactions [63]:

M + Cl^−^ → MCl + H^+^ + e^−^ (7)

MCl → MCl^+^ + e^−^ (8)

MCl^+^ → M^+^ + Cl^−^ (9)

Furthermore, the corrosion resistance of the 316LV austenitic stainless steels is improved by 2–3% of molybdenum. The presence of this element as an alloying element reduces the number and the size of nucleation and metastable pits [2]. Therefore, in future research, the addition of molybdenum is planned to improve the corrosion resistance of the tested material. 

## 5. Conclusions

The nickel-free austenitic stainless steel with the composition of Fe–18%Cr–12%Mn–0.5%N was prepared by MA followed by the sintering or HIPping consolidation methods. Microstructure observations, phases identification, mechanical properties, and corrosion tests were performed. The main conclusions are summarized as follows:

(1) After 40 h of MA of the powder, the austenite phase appeared and the two-phase structure (α-Fe and γ-Fe) was characterized by XRD.
(2) The nitrogen atmosphere and Mn_4_N compound powder addition allowed to obtain an fcc phase structure, confirmed by the XRD analysis, after 90 h of MA despite not raising the process parameters.
(3) The MA powders after sintering at the temperature of 1150–1200 °C for 2 h in nitrogen has about 1.0% of N_2_ content and the austenitic structure with Cr_2_MnO_4_ oxides, with 85–88% of the theoretical density depending on the sintering temperature applied. The sample sintered at 1200 °C in nitrogen had a slightly higher density and hardness in comparison to the sample sintered at 1150 °C.
(4) The HIP method allowed to obtain the material with a relative density of ~97%. The different cooling rate applied after HT (water quenching or furnace cooling) strongly affected the properties of the HIPped material.
(5) The material with furnace cooling had the highest density, better ductility, and higher corrosion resistance in the Hank’s solution in comparison to the other specimens tested. However, in this material, the Mn content fluctuates with the distance from the surface of the sample to the core, causing porosity and Mn depletion related to the high vapor pressure of this element in a vacuum.
(6) The potentiodynamic curve of the heat treated and furnace cooled HIPped material was similar to 316LV, but the corrosion potential value was lower than 316LV. The corrosion current of the HIP+HT–FC and 316LV samples was similar. All of the above indicated that the HIPped and heat treated, furnace cooled material obtained in this work had similar corrosion properties to austenitic stainless steels (316LV) commonly used in medicine.

## Figures and Tables

**Table 1:** Internal lattice d-space and nitrogen content of the Fe–18%Cr–12%Mn powder and literature data for Fe–18%Cr–11%Mn elemental powders of Fe, Cr, and Mn ball milled in nitrogen.

Milling Time (h)	Fe–18%Cr–12%Mn Powder		Fe–18%Cr–11%Mn Powder *	
	d (111) (nm)	Nitrogen (wt.%)	d (111) (nm)	Nitrogen (wt.%)
20	0.20310 ± 0.0124	0.23 ± 0.011	-	0.32
40	0.20308 ± 0.0112	0.35 ± 0.013	0.20685	0.41
60	0.20377 ± 0.0098	0.48 ± 0.021	0.20712	0.5
80	0.20603 ± 0.0095	0.72 ± 0.016	0.20739	0.62
90	0.20966 ± 0.0086	1.01 ± 0.025	-	-
100 *	-	-	0.20807	0.65

* Literature data [14].

**Table 2:** Summary of statistical parameters for tested powders.

Statistic Parameter of the Powders	Powder after Different Time of MA	
	1 h	90 h
De Bruckere Mean D[4,3] (µm)	89.6 ± 2.2	51.9 ± 1.2
Median D50 (µm)	92.2 ± 1.6	38.9 ± 0.9
Mode Mo (µm)	109.1 ± 3.4	34.3 ± 1.4

**Table 3:** The density, hardness and grain size data of the sintered specimens.

Material	Density (kg/m^3^)	Relative Density (%)	µHV0.2 (MPa)	HV10 (MPa)
Sintered at 1150 °C	6250 ± 26	85.2	3413 ± 235	2697 ± 176
Sintered at 1200 °C	6450 ± 31	87.8	3040 ± 117	2893 ± 156

**Table 4:** Bulk chemical composition of the tested materials.

Materials	Element (%)					
	Cr	Mn	N	O	C	Fe
Powders after 90 h of MA	18.42 ± 1.52	13.57 ± 0.57	1.01 ± 0.025	0.48 ± 0.13	0.0821 ± 0.0021	Bal.
As-HIPed	18.31 ± 0.55	12.07 ± 0.31	0.806 ± 0.071	0.421 ± 0.012	0.0601 ± 0.0012	Bal.
HIP+HT–FC	18.03 ± 0.77	11.98 ± 0.24	0.451 ± 0.063	0.210 ± 0.022	0.0575 ± 0.0019	Bal.
HIP+HT–WC	18.24 ± 1.09	12.02 ± 0.21	0.543 ± 0.055	0.311 ± 0.018	0.0562 ± 0.0022	Bal.
Sintered at 1150 °C	17.96 ± 0.61	12.24 ± 0.18	1.023 ± 0.064	0.512 ± 0.026	0.0675 ± 0.0016	Bal.
Sintered at 1200 °C	18.11 ± 0.97	11.95 ± 0.22	0.918 ± 0.082	0.498 ± 0.023	0.0662 ± 0.0018	Bal.

**Table 5:** The density, hardness, and grain size data of the tested specimens.

Material	Density (kg/m^3^)	Relative Density (%)	µHV0.2 (MPa)	Grain Size (µm)
As-HIPed	7430 ± 11	96.5	4786 ± 108	1.76 ± 0.35
HIP+HT–FC	7448 ± 19	96.8	2972 ± 69	2.47 ± 0.49
HIP+HT–WC	7426 ± 21	95.8	5688 ± 843	2.35 ± 0.42

**Table 6:** Tensile properties of as-HIPped and heat treated specimens.

Mechanical Properties	As-HIPped	HIP+HT–FC	HIP+HT–WC
E [GPa]	194		
UTS [MPa]	934 ± 11	938 ± 8	975 ± 10
ε_t_ [%]	0.50 ± 0.02	3.51 ± 0.13	0.41 ± 0.01
YS_0.2_ [MPa]	-	741 ± 10	-

**Table 7:** The main parameters of corrosion process of HIPped samples.

Material	*E_cor_* (mV)	*R_p_* (ohm)	*b_a_* (V)	*b_c_* (V)	*I_cor_* (uA/cm^2^)
HIP	−495.4 ± 23.3	6740 ± 155	0.0785 ± 0.0026	0.0610 ± 0.0021	2.090 ± 0.012
HIP+HT–FC	−72.4 ± 2.6	473790 ± 1236	0.0624 ± 0.0017	0.0407 ± 0.0013	0.023 ± 0.004
316LV	−139.6 ± 11.5	532300 ± 1453	0.0497 ± 0.0012	0.0331 ± 0.0011	0.016 ± 0.003
